# How decentralisation influences the retention of primary health care workers in rural Nigeria

**DOI:** 10.3402/gha.v8.26616

**Published:** 2015-03-03

**Authors:** Seye Abimbola, Titilope Olanipekun, Uchenna Igbokwe, Joel Negin, Stephen Jan, Alexandra Martiniuk, Nnenna Ihebuzor, Muyi Aina

**Affiliations:** 1 National Primary Health Care Development Agency, Abuja, Nigeria; 2 School of Public Health, University of Sydney, Sydney, Australia; 3 The George Institute for Global Health, Sydney, Australia; 4 Solina Health, Abuja, Nigeria; 5 Dalla Lana School of Public Health, University of Toronto, Toronto, Canada

**Keywords:** human resources for health, retention, rural, primary health care, governance, decentralisation, community health committees, Nigeria

## Abstract

**Background:**

In Nigeria, the shortage of health workers is worst at the primary health care (PHC) level, especially in rural communities. And the responsibility for PHC – usually the only form of formal health service available in rural communities – is shared among the three tiers of government (federal, state, and local governments). In addition, the responsibility for community engagement in PHC is delegated to community health committees.

**Objective:**

This study examines how the decentralisation of health system governance influences retention of health workers in rural communities in Nigeria from the perspective of health managers, health workers, and people living in rural communities.

**Design:**

The study adopted a qualitative approach, and data were collected using semi-structured in-depth interviews and focus group discussions. The multi-stakeholder data were analysed for themes related to health system decentralisation.

**Results:**

The results showed that decentralisation influences the retention of rural health workers in two ways: 1) The salary of PHC workers is often delayed and irregular as a result of delays in transfer of funds from the national to sub-national governments and because one tier of government can blame failure on another tier of government. Further, the primary responsibility for PHC is often left to the weakest tier of government (local governments). And the result is that rural PHC workers are attracted to working at levels of care where salaries are higher and more regular – in secondary care (run by state governments) and tertiary care (run by the federal government), which are also usually in urban areas. 2) Through community health committees, rural communities influence the retention of health workers by working to increase the uptake of PHC services. Community efforts to retain health workers also include providing social, financial, and accommodation support to health workers. To encourage health workers to stay, communities also take the initiative to co-finance and co-manage PHC services in order to ensure that PHC facilities are functional.

**Conclusions:**

In Nigeria and other low- and middle-income countries with decentralised health systems, intervention to increase the retention of health workers in rural communities should seek to reform and strengthen governance mechanisms, using both top-down and bottom-up strategies to improve the remuneration and support for health workers in rural communities.

The shortage of health workers in rural areas continues to be a growing concern globally (1–3). In 2010, the World Health Organization (WHO) recommended a list of educational, regulatory, financial and supportive interventions to promote retention of health workers in rural areas (4). These generic interventions were proposed for policy-makers to combine several of them and implement them as a package, selecting the most effective mix given the context in each setting. Although the government of Nigeria has yet to implement the WHO recommendations, Nigeria's strategy on human resources for health (5) also indicates that efforts to improve the retention of rural health workers should be based on in-depth analyses of contextual factors. As reflected in the 2010 WHO recommendations, retention of rural health workers is influenced by a range of considerations including salary and working conditions, access to in-service training, career advancement opportunities, targeted admission of students, and recruitment of health workers from rural areas and performance management systems (4). Using information on these and other factors, however, requires taking into account how they may contribute to retention of rural health workers within different health systems (6, 7). In countries like Nigeria where the health system is decentralised, it is important to understand how decentralisation influences the retention of health workers in rural communities (8).

In Nigeria, successive national constitutions (starting in 1979, then in 1989, and most recently in 1999) have all prescribed a decentralised structure of governance. This governance structure includes devolution of the responsibility for financing and managing local primary health care (PHC) facilities to sub-national governments (see Box 1 for definitions of different forms of decentralisation) (11). PHC refers to preventive or curative health care provided in a community setting to people making an initial approach to the health system for advice, tests, treatment, or referral to specialist care (12). Formal health-care services in rural Nigeria are largely provided through public sector PHC facilities, and they often reach far into remote parts of the country (13). Each community is part of a local government area, which are administered by local (district) governments. These local governments together with state (provincial) governments provide logistics and human resources for health to implement PHC, while the federal (national) government provides policy, oversight and technical support for PHC. However, the decision on which sub-national (state or local) government takes primary responsibility for PHC depends on the arrangement in each state (14, 15). Typically, states are in charge of recruiting health personnel to work in PHC facilities owned and operated by local governments (16). Although state governments retain this responsibility for senior personnel (such as nurses, midwives, doctors, and senior community health workers), they delegate the responsibility for initiating the process of hiring more junior personnel (including junior community health workers) to local governments, although final decisions are taken at the state level (17).

***Box 1.* Definitions^a^ of types of decentralisation in relation to primary health care (PHC)**

*Decentralisation* is a system of governance in which the power, authority, resources, and responsibility for PHC service delivery are transferred from a central government to actors and institutions at the periphery. With the governance closer to the people, this transfer of responsibilities allows for local initiative, input, and control. Forms of decentralisation include devolution, deconcentration, and delegation.

*Devolution* refers to the transfer of responsibility for PHC to autonomous administrative structures or governments. In principle, these structures, such as local, municipal, state, and provincial governments are independent of the central government with respect to a defined set of responsibilities. But in practice different contextual factors may limit or enhance the capacity of peripheral governments to function.

*Deconcentration* refers to a central government handing over some of its authority for PHC to the peripheral offices of the administrative structure of the central government, such as field offices of its ministry responsible for health. These offices have some discretion to manage health-related activities without constant recourse to central government officials.

*Delegation* refers to the transfer of defined managerial or administrative responsibilities to institutions outside the administrative structure of a central government. These institutions include semi-autonomous agencies such as a regulatory commission or a community health committee, and they can be indirectly controlled by the central government ministry responsible for health.

^a^These definitions were adapted from Mills et al. (9) and Frumence et al. (10).

Public sector financing in Nigeria is tied to national funds, which are shared among the tiers of government according to a formula that gives approximately half of the funds to the federal government, a quarter to the 36 states that make up Nigeria, and the other quarter to the 774 local governments in Nigeria (18). The federal government is largely responsible for tertiary care, state governments for secondary care, and as mentioned previously, local governments typically run PHC. However, because allocation to local governments is channelled through state governments (in line with the 1999 constitution), states have constitutional control over the amount of funding that reaches local governments (19). Channelling local government funds through states had earlier been prescribed in the 1979 constitution, but was abolished in the 1989 constitution to strengthen local governments. The system was later reinstated in the 1999 constitution because of concerns that direct transfer of funds fostered corruption at the local government level (11). However, this situation in which responsibility is devolved to local governments without guaranteed matching financial support results in varying patterns of health outcomes, depending on how communities are able to influence the supply and demand of PHC services (20, 21).

National health policy is to institutionalise community engagement in PHC; responsibility for this engagement is delegated to community health committees, but without financial support for their activities (22). The committees are established through a participatory approach to assist communities in identifying their PHC challenges and finding appropriate solutions. Committee members include respectable members of the community, primary and secondary school head teachers, the health worker in charge of the health facility, and representatives of traditional, voluntary, religious, women, youth and health-related occupational groups – informal health-care providers such as traditional healers, traditional birth attendants, and patent medicine vendors (local chemists and drug shops). The chair, secretary, and treasurer of the committee are appointed by members of the committee, and they are expected to meet at least once every month (23). Communities with PHC facilities in Nigeria typically have these community health committees as an additional level of PHC governance (22, 24). These committees (also known as ward or village development committees) are part of the decentralised process of PHC governance in Nigeria. And their responsibility for community engagement and the consequences of that responsibility are part of non-government contributions to decentralised PHC governance in Nigeria (22).

The shortage of health workers is worst at PHC facilities in rural communities where half of Nigeria's 170 million population live (25). Perhaps because PHC is often the only form of formal health service available to rural populations, the shortage of rural PHC workers is also associated with worse health outcomes in rural communities compared to urban areas (26, 27). Although Nigeria has no routine systematic data on availability, distribution, and trends in human resources for health, a national survey conducted in 2005 showed higher health-worker attrition rates in rural compared to urban health facilities (28). Attrition of doctors from rural areas was triple the rate from urban areas, while for nurses it was double the rate. In addition, the attrition of doctors and nurses was much higher at the PHC level compared to secondary care (run by state governments) and tertiary care (run by the federal government) such that in the public sector, only 19% of doctors and 31% of nurses worked at the PHC level (28). With the exception of community health workers (a lower cadre of health workers trained for 2–3 years specifically to work at the PHC level, 91% of whom worked at the PHC level), health workers in rural communities in Nigeria tend to seek posting to urban areas or leave to work in secondary or tertiary care.

In 2013, as part of efforts to domesticate the WHO recommendations in Nigeria, we obtained qualitative data on the perspectives of PHC stakeholders in Nigeria on retention of rural PHC workers. In this report, we focus on how decentralisation influences the retention of PHC workers in rural Nigeria. Previous studies on retention of rural health workers (29–33) and on the impact of decentralisation on health workers (8) have been based on the perspectives of health workers and managers, often excluding the beneficiaries of health services: the community. In addition, previous studies of the impact of decentralisation on health workers (8) and the 2010 WHO recommendations on retention of rural health workers (4) did not systematically explore how decentralisation influences the retention of health workers in rural communities – even though there is an increasing trend toward adopting decentralisation reforms of health system governance among low- and middle-income countries (LMICs) (8). In this paper, we use multi-stakeholder perspectives to explore how decentralisation influences the retention of PHC workers in rural communities in Nigeria.

## Methods

This qualitative study was conducted between April and July 2013, and the findings presented in this paper are based on in-depth interviews (IDIs) and focus group discussions (FGDs). In this report, we followed the requirements in the consolidated criteria for reporting qualitative research (COREQ) for interviews and focus groups (34) for information on the research team, study design and data analysis reported as indicated.

### Study setting

The study was conducted in six states in Nigeria, three in northern Nigeria (Kaduna, Nasarawa, and Benue) and three in southern Nigeria (Lagos, Bayelsa, and Abia). These states were chosen for their geographic spread, encompassing the major geopolitical and ethnic groups across the north and south of Nigeria. Each of the six states has an average of about 18 local governments. Six rural communities were selected from different local governments for the study through a purposive sampling process to ensure a broad range of perspectives are represented. All the communities included in this study had community health committees, although this was not a criterion for inclusion. In Nigeria, a minimum of 19 health workers are expected to staff a standard PHC facility, comprising one medical doctor, four health workers in the nurse-midwife category, ten in the community health worker category, one each of pharmacy and laboratory technician, and one each of medical records and environmental officers (23). However, having a full complement of health workers in a PHC facility depends on availability. Therefore, health workers in the nurse-midwife and community health worker categories constitute the mid-level health workers (with at least 2–3 years of post-secondary school health-care training), who undertake tasks typically carried out by medical doctors, such as clinical or diagnostic functions. To make up for the gaps in health workers with higher qualifications, mid-level health workers are increasingly used to deliver services autonomously, particularly in rural communities of LMICs (3).

### Study participants

The study participants were purposively selected to ensure that participants have the potential to provide rich, relevant, and diverse information on the research question. In each of six states, we conducted three FGDs with groups of PHC workers and three FGDs with groups of community members. In addition, we conducted IDIs with PHC workers, community members, and PHC managers working at local, state, and federal tiers of government (9 with community leaders, 8 with health committee members, and 15 with PHC managers). Each FGD involved 8–10 participants and lasted approximately 90 min, and each interview lasted approximately 60 min. We included as study participants all formal and full-time PHC workers involved in direct health-care provision such as nurses, midwives, community health workers, counsellors, and environmental health, laboratory and pharmacy personnel. Support staff such as cleaners and security guards were excluded, as were potential participants who could not communicate in any Nigerian language or who declined to sign consent forms. Given limited resources and time for this study, we also excluded all potential participants who were less than 18 years old because of logistic and ethical concerns associated with obtaining consent from and interviewing minors or involving them in group discussions.

### Study instruments

We developed semi-structured IDI and FGD questions and prompts to explore issues affecting the retention of rural PHC workers: financial incentives, career advancement opportunities, working and living conditions, community acceptance and support, the physical and social attributes of communities, and the personal and social attributes of health workers. If the respondent cited issues related to decentralisation as a reason for lack of retention, the study instrument provided scope to probe how and why and to proffer suggestions to improve retention of rural PHC workers. The findings presented in this paper are limited to those related to decentralisation.

### Data collection and management

Interviews and discussions were conducted by six trained researchers in pairs. They were staff and consultants to the National PHC Development Agency in Nigeria, selected for their ability to speak the local languages of their respective study states. They were briefed for the purpose of this study by two of the authors (SA and MA). Researchers and participants met for the first time during the study, but there were prior telephone contacts to schedule data collection. The study objectives were explained to the participants and confidentiality was assured. Participants agreed at the beginning of each FGD to maintain confidentiality within the group by not discussing outside the group individual opinions raised by others during discussions. Interviews and discussions were conducted within health facility premises or an open space nearby. By the time we had conducted 32 IDIs in total and six FGDs in each state, participants were no longer presenting new issues; at this point researchers agreed that data saturation had been reached. There were no repeat interviews or discussions. When required, the data was translated to English by the researchers who conducted the interviews. The IDIs and FGDs were tape-recorded and data were subsequently transcribed and transferred to Microsoft Excel to aid analysis.

### Data analysis

We conducted directed content data analysis (35), by coding and categorising patterns in the data while taking into account the multi-level governance of PHC in Nigeria (22). Two authors (SA and TO) read the transcripts independently and used bottom-up coding to categorise issues related to health system decentralisation emerging as contributors to retention of rural PHC workers. Disagreements in coding and discrepancies in interpretation were discussed and decided by consensus. Phrases or quotes that most accurately expressed or illustrated the categories under each theme were then identified.

### Conceptual framework

Our analysis drew on existing literature and conceptual frameworks and their applications, linking health sector reforms (such as decentralisation) to human resources management. We considered three potential influences on the motivation and retention of health workers: 1) government or organisational influences; 2) community influences; and 3) intrinsic health worker influences (i.e. how health workers respond to organisational and community influences) (22, 36). In addition, we took into account three factors that may link decentralisation to organisational and community influences: 1) which tier of government takes responsibility for decentralised functions; 2) how clearly defined are the responsibilities for these functions within and between tiers of government; and 3) what technical and financial capacity and resources are available at each tier of government to perform these functions (8, 22, 37). We further identified the functions that have a bearing on motivation and retention broadly as the following: 1) recruiting and deploying health workers; 2) paying the salary of health workers; 3) supporting and managing health-worker performance; and 4) providing resources and infrastructure for optimal performance (4, 8, 22, 36, 37).

Our analysis was also informed by a previous framing of factors influencing the response of health workers to government or organisational influences and to community influences as ‘push’ and ‘pull’ factors (38). In our analysis, push factors are those that encourage health workers to leave their rural PHC post for an urban community or another level of the health system. They often mirror pull factors, which are factors that attract the movement of PHC workers to urban communities or higher levels of care. There is a second set of factors: ‘stick’ and ‘stay’ factors (38). In this study, stick factors consist of reasons why health workers do not leave rural communities in spite of compelling push factors. Stay factors are those that prevent health workers from leaving urban communities. Given that we collected data from rural communities, our analysis only considered push and stick factors. Our analysis was further informed by theories that have been previously used to further understand the push-pull-stick-stay factors (7): the neoclassical theory which suggests that the factors are influenced largely by the motive to maximise income and employment opportunities (39); and the behavioural theory which suggests a more complex decision-making process encompassing other forms of satisfaction that health workers derive from their work or posting (40).

### Ethics

Ethics approval for this study was provided by the National Health Research Ethics Committee of Nigeria. Participation in the study was entirely voluntary and based upon the participant signing a written informed consent form. In line with the terms of consent to which participants agreed, the data for this study are not publicly available and all participants have been de-identified, by removing information on name, gender, cadre, community, and local government of participants.

## Findings

The themes that emerged to characterise how decentralisation influences the retention of health workers in rural communities were either attributable to fragmentation of responsibility among tiers of government or to community engagement in PHC delivery as a result of the activities of community health committees. The themes attributable to fragmentation of responsibilities constitute ‘push’ factors, whereas the themes related to community engagement are ‘stick’ factors (see Box 2 for the categories under each theme).

**Box 2:** How decentralisation influences the retention of primary health care (PHC) workers in rural Nigeria

Fragmentation of responsibilities in the health system among tiers of government	The salary to PHC workers is paid irregularly due to inefficiencies in the chain of funds transfer from one tier of government to another. Lower salaries at the PHC level compared to secondary and tertiary care because better funded tiers of government are responsible for the higher levels of health care.
Community engagement in PHC through the community health committees	Improved uptake of PHC services increases job satisfaction among health workers, leading to reduced absenteeism, which implies retention. Social and financial support for health workers by community members increases job satisfaction and motivate them to stay in rural communities. Co-financing and co-managing PHC facilities by community members ensures they function optimally, thereby increasing job satisfaction of health workers, which may lead to retention.

### Fragmentation of responsibilities in the health system

There was a sense that the most significant challenge to the welfare and motivation, and therefore retention, of PHC workers in rural communities was irregular and uneven salaries. Participants described push factors attributable to two forms of fragmentation in the health system, even though these are not peculiar to rural communities. First is the fragmentation of responsibility for PHC among the three tiers of government. This fragmentation results in irregularities in the payment of the salary of PHC workers resulting from the long chain of transfer of funds from the federal government. Second is the fragmentation of responsibility for health care, so that the federal government is responsible for tertiary care, the states for secondary care, and the much weaker local governments for PHC. Thus lower salary is a push factor for PHC workers to the secondary and tertiary levels of care, which are typically in urban communities. These two forms of fragmentation combine to make working at the PHC level unattractive to health workers. However, while the neoclassical theory of maximising income explains the concerns about salaries, these concerns are made worse because health workers at other levels of care are better off – a situation that is better explained by the behavioural theory because the push factors relate to positional concerns about the relative amount and regularity of income among perceived peers at other levels of care, leading to low motivation and attrition among PHC workers.

#### Irregular salary payments

Health workers spoke strongly about irregularities in the payment of their salary as a reason why they would prefer to work at other levels of care. For example, a PHC worker in Bayelsa said, ‘Some of us haven't been paid for months and this has brought down our morale. In the state [secondary care] such things don't come up. We feel we are cheated in the local government. This makes us want to leave because it doesn't happen in the state’. This statement suggests that it is not only the absolute situation of salaries that matters, but also how it compares to the situation of perceived peers. Participants in this study had a view that responsibility for PHC is fragmented and this makes it difficult to know whom to hold accountable for irregular payment of salaries. In the words of a PHC worker in Bayelsa expressing frustration about this difficulty, ‘This problem [of prompt payment of salaries] comes from all over, from state, federal, and local governments. We don't even know who is supposed to pay us’. This confusion makes it possible for one tier of government to blame failure on another tier of government. In one example, a state PHC manager in Benue blames the local government, expressing the inability of the state to intervene in the failure to pay the salaries:

There are times health workers in PHC are not paid salaries for two to three months and this problem usually comes from the local government and there is really nothing my [state level] department can do about it. The management of PHC is under the purview of local governments and they have not really been doing anything to address these challenges.

In addition, a local government PHC manager in Benue proposed a solution to irregular salary payments by saying ‘the federal government should increase the allocation to local governments through the state government or better still pay the local governments directly. This will help resolve the problem of delay in payment of salaries’. However, some participants identify local governments as being responsible for delays in the payment of salaries as a result of corrupt practices. And others mentioned that salaries are not paid by local governments because state governments withhold funds allocated to local governments. There was also an impression that salary delays resulted from the long chain of transfer of funds, first from the federal to state governments and subsequently the state to local governments. One PHC worker in Benue suggested that their salaries ‘should come right from the federal government so [that] no one can tamper with it’. This is because in compliance with the Nigerian constitution, local government funds are channelled through accounts held by state governments. But there were challenges of irregular salaries of PHC workers in the 1990s when local government funds were transferred to them directly (24), indicating that lapses are due to broader issues of accountability.

#### Uneven salary between levels of care

Health workers also spoke strongly about the difference between their salary and that of their counterparts working for the state and federal governments at secondary and tertiary levels of care. In Lagos, a PHC worker said ‘We went to the same schools and have the same certificate as the health workers at the state and federal levels. Then why do we have different salaries?’ This statement suggests that it is not only the absolute amount of salary that matters, but also how it compares to the salary of perceived peers. Participants therefore proposed that state governments should take primary responsibility for PHC, echoing ongoing policy advocacy (since 2010) by the federal government for states to establish a streamlined governance mechanism in which states instead of local governments take primary responsibility for all aspects of PHC service delivery. In this proposal, rather than being administered by autonomous but poorly funded local governments (devolution), PHC will be administered by operational sub-units of the state government superintending over PHC in different local governments (deconcentration) (16). In Lagos, where that is already the case, a PHC manager said that paying PHC workers’ salaries equal to those of health workers employed by the state government in secondary care ‘has helped us retain our PHC workers at the local government’. The lower salary levels of PHC workers may result from low budget availability at the local government level. But PHC workers feel they are unfairly treated, as health workers at other levels get paid more because of the fragmentation of responsibility for health care.

### Community engagement in PHC

Participants also emphasised how the actions of communities constitute stick factors in influencing the retention of rural PHC workers. The stick factors overcome push factors such as irregular salary and lack of job satisfaction due to low uptake of services, lack of health facility infrastructure, social network, and accommodation. These ‘stick’ factors include the job satisfaction PHC workers derive from the increased uptake of services that results from the activities of community health committees – this leads to reduced health-worker absenteeism, which for many stakeholders is tantamount to retention. Participants also gave examples of other stick factors such as the efforts of community health committees (often in response to government failure) to ensure that health workers stay to work in their community. These efforts include providing support to PHC workers and co-financing and co-managing PHC services to ensure that PHC facilities are functional even in the absence of government support. These stick factors improve job satisfaction and can motivate PHC workers to stay and work in rural communities in spite of the push factors related to irregular salary. This suggests that considerations of other forms of satisfaction (behavioural economics theory) can possibly trump that of maximising income (neoclassical economics theory). However, another stick factor for retention may be the limited options for employment elsewhere for the community health workers who are specifically trained to work at the PHC level.

#### Community uptake of PHC services

Participants described how preference for informal health providers in rural communities (such as traditional healers, traditional birth attendants, and chemists or drug shops) leads to low demand for formal PHC services. This low demand for services discourages PHC workers, leading to absenteeism or a decision to leave. In some cases, preference for informal providers stems from previous experience in which people could not access care due to costs or because of absenteeism. A local government PHC manager in Abia said, ‘There are traditional healers and birth attendants in the communities. They are alternatives to the health facilities. People go to them for health care instead and this is very discouraging to the health workers at the facilities’. In Bayelsa, a community health committee member said a reason for absenteeism is that ‘The workload is too small. Not that people do not fall sick, but some villagers go straight to the chemist and take care of themselves at times because the nurse is not available’. And a PHC worker in Abia excused absenteeism by saying, ‘If their turnout is great we will have the zeal to be here to work. When we come in and there is no one coming, nobody is responding, then you start feeling bored. You will be reluctant to come to work because there is nothing to do. You can stay here for up to a week without seeing a patient’. Ensuring that people are able to use PHC services was seen by community representatives as a way of supporting PHC workers. As one community health committee member in Lagos said: ‘We [the health committee] sometimes pay for drugs for the patient to encourage the health workers to stay back at the PHC facility and work’.

#### Community support for PHC workers

Participants described ways in which individual community members and groups support PHC workers, thereby potentially contributing to their retention. Communities provide support to PHC workers in various ways, for example by helping them find a good place to stay, as one community religious leader in Benue said: ‘We try to look for where [it] is conducive for them to stay. We assist them to look for the house and they pay the rent themselves’. Accommodation was identified by one PHC worker in Lagos as ‘the biggest factor that affects retention of health workers’ in rural PHC. Communities also make PHC workers feel at home in order to encourage them to stay, for example by visiting them and calling them on the phone. Community health committee members in two communities in Abia said, ‘We come to the facility to keep them company’ and ‘We call them [on their mobile phone] so they don't feel lonely and bored’. In Nasarawa, a community health committee member said, ‘We find out if they need anything like cleaning of their houses’. This was confirmed by many PHC workers; for example, one in Kaduna said: ‘Yes, they usually sympathise with us and ask us about our problems. The little support we get from them takes us a long way and this is the reason why we are staying’. People in different communities also support PHC workers financially, sell groceries to them on credit, and give them foodstuffs. This was also confirmed by many PHC workers. One in Bayelsa said ‘If you're short of money, they can let you take things then pay later because you are trusted’. Another in Kaduna said, ‘The community gives us money and foodstuff. They even lend us money. I even want to go. They are the reason we are staying’. Unsurprisingly, PHC workers with lower expectations (especially community health workers) responded more to these forms of support. However, there was an instance in Benue where a community health committee raised funds to hire a doctor to work part time at their PHC facility.

#### Co-financing and co-managing PHC facilities

In response to the question on what the communities feel they can do to retain health workers, participants often responded by giving examples of how communities co-finance and co-manage PHC facilities so that health workers will want to stay and work there. This effort is often in order to assuage effects of government failure to support PHC. In one of the many instances of this, a community health committee member in Kaduna said, ‘When things spoil [in the PHC facility] we try and give money to repair them. But when it is too much for us, we write to the local government to do it. Sometimes we write and write about our complaints but it is not always successful’. In Nasarawa, another community health committee member responded by saying, ‘Where the government fails to pull through, the community members meet, to work together to provide amenities’ in the PHC facility. Others in Benue described how ‘we help clear the surroundings’, and ‘when there is a building problem, we repair it’. In Kaduna, one community member said ‘We provide them [PHC workers] with minor working materials such as brooms and buckets’. In Lagos, another said, ‘We helped fix the doors in order to improve the security’. And in Abia, one said, ‘We pay for [electricity] generator fuel’. In another example, a community contributed funds to build a place for PHC workers to stay. The community leader said, ‘Have you seen those [concrete] blocks? It's our money we are using for this. Now we have gotten 1,000 blocks to build the staff quarters for health workers. We started 5 months ago. We think this will make the staff stay back’. This is consistent with a 2003 survey in Kogi State, Nigeria, which showed that community health committees were the main source of support for building maintenance in 57% of 140 PHC facilities (24).

However, there were also communities where PHC workers discussed unmet expectations of support from the community perhaps because, as explained by one community member in Kaduna, ‘We feel the government is supposed to do everything for them. We don't think whatever we give will do anything for the health workers’. In response to notions such as this, participants stressed the need for responsive communities with community health committees that can provide support for PHC workers. For example, a state PHC manager in Benue proposed formal requirements of community support for PHC workers through health committees, which ‘should be mandated to source for accommodation for PHC workers posted to their communities’. Likewise, a local government PHC manager in Benue said, ‘Communities should be made to contribute money to further support the provision of drugs and other minor equipment at the facilities to motivate the PHC workers’. But there are limits to such expectations given the low level of income in many communities and because, as a community member in Nasarawa said, ‘We cannot do anything for them [PHC workers] because we also face some of the challenges they are facing, like inadequate infrastructure in the community’. It is also noteworthy that participants did not mention that monitoring and supervision of health workers by community members and representatives led to or may lead to reduced absenteeism. These limitations suggest that the presence of a health committee in a rural community is no guarantee that there will be successful collective action to reduce absenteeism or support PHC workers to increase their retention in the community.

## Discussion

The findings of this study provide additional information on the retention of rural health workers, with implications for policy and practice in LMICs with decentralised health systems. In Nigeria, decentralisation of health services leaves PHC governance to the weakest tier of government (local governments). Therefore health workers prefer working at the secondary and tertiary levels of care (run by states and the federal government), where salaries are better and more regular, or in urban PHC facilities, where living conditions are better (41). In addition, because responsibility for PHC is shared among different tiers of government, payment of salary of PHC workers tends to be irregular as one tier of government can blame failure on another tier of government. In addition, our analysis shows that community health committees can play an important role in retaining health workers in rural communities by supporting health workers, given that governments tend to fail in their responsibility to pay salaries regularly and to provide other basic essentials for health workers in rural communities. Committees can also play a vital role in generating demand for PHC services, which leads to job satisfaction for health workers and in some instances can help increase PHC worker retention. Through the committees, communities also advocate to governments to support PHC, and when that fails they can co-finance and co-manage PHC services.

The findings are in keeping with the results of previous studies in LMICs. For example, studies in China (42) and Tanzania (43) showed that without matching decentralisation with mechanisms for retaining health workers in rural areas, better qualified personnel tend to leave lower level health facilities in rural areas for better remunerated employment in higher level and urban facilities. Likewise, evidence from Uganda and South Africa suggest that where salaries or benefits are determined locally, variations in remuneration may result in movement of health workers away from rural areas to where governments are able to provide better incentives (8). In addition, previous studies on the impact of decentralisation on health workers suggest that delays in disbursement of funds from the national government (10) and lack of technical capacity and financial resources to manage human resources for health at lower levels of government is a common feature of decentralisation in LMICs (8). In a multi-country stakeholder perspective study (44) and another study in rural Nigeria (45), both on the retention of lay health workers in rural communities, support by community health committees was identified as an important contributory factor to retention. Our study extends this literature by demonstrating that community health committees can also play important roles in the retention of formal health workers in rural communities. Indeed, previous studies of the economics of staff motivation in other disciplines have shown that stick factors such as social support in the work environment can be more important than push factors such as low pay (46). Given the link between motivation and retention in rural communities (47), the perception among rural health workers of being unfairly treated, including positional concerns about their relative income, leads to low motivation and attrition (48).

Improving the retention of rural health workers in decentralised health systems may require strategies to strengthen the technical and financial capacity of local governments to plan and implement PHC in rural areas, with clearly defined responsibilities and accountability mechanisms among tiers of government. In addition, improving the retention of rural health workers may require interventions to strengthen structures such as community health committees. The literature on community participation in PHC suggests that in many LMIC settings communities often require government support to ensure effective engagement (49). This support may include government policies that promote and support the engagement of communities in their own health systems (50). For example, educating communities about the role they can play in retaining their health workers and helping them do so by supporting the activities of community health committees through grant schemes, for example, may help increase retention of rural health workers (51). In addition, providing these committees with information on the resources and responsibilities of different tiers of government can improve government accountability by allowing communities to target their advocacy more appropriately (52). However, implementing these bottom-up community initiatives at a national scale requires the flexibility to engage with local issues and adopt local solutions in different settings within a country (44).

The case for decentralising public services rests on the expectation that governments that are closer to communities (such as local/district governments) will also be more responsive to communities; these communities will in turn be able to better articulate their needs to proximate local government officials and hold them to account. This argument assumes that higher tiers of government such as states (provincial) and federal (national) governments will be willing to provide adequate financial resources and devolve full power and responsibility to local governments (53). But as shown in this and previous analyses of decentralisation in LMICs, often these assumptions do not hold true; decentralisation often does little to improve public service delivery (54). Therefore, in LMICs where health system governance is weak, interventions to strengthen local governments and support for community engagement should be incorporated among interventions to improve the retention of rural health workers (4, 55). However, implementing these interventions requires investigating how to design decentralisation without the unintended effect of making the system susceptible to failure at one or more tiers of government. Further studies should explore factors that influence collective action for PHC in a community and tease out the contextual factors that contribute to community support for health workers. Studies should also explore how other potential stick factors may influence retention: for example, having free onsite accommodation within rural PHC facilities or supporting health workers to have their family reside with them during rural postings.

In line with existing evidence, this study demonstrates that a mix of financial and non-financial factors constitute the factors necessary for retention of PHC workers in rural communities. But the capacity to intervene successfully depends on context, not least the context of health system governance. For example, introducing stick factors such as accelerated career progression for health workers in rural communities or a dedicated rural career pathway may foster the retention of rural health workers (38). But in decentralised systems fragmentation of career structures may limit the effectiveness of such initiatives. In addition, addressing other socio-economic push factors such as poor living and working conditions and lack of accommodation may require concerted government efforts, which may also be limited by fragmentation – so is capitalising on potential stick factors such as targeting students from or with family and social ties in rural communities. It is important, therefore, that LMICs that are just embarking on decentralisation reforms specifically explore how decentralisation may influence the retention of rural PHC workers. Lessons from the Nigerian experience include the need to avoid unnecessary fragmentation and poorly defined lines of responsibility and to ensure that community governance structures are supported as an integral part of decentralisation reforms.

There are two potential limitations to this study. One is that all the communities had functional community health committees. Although a previous study suggested that in Nigeria the majority of rural communities with PHC facilities have community health committees (24), it is important that our results are interpreted with caution in areas where these committees have not taken off or do not function as well. Secondly, our study did not include politicians and urban communities. But the health managers we included oversee both rural and urban PHC facilities, and so their perspectives potentially reflect realities beyond rural communities. However, future studies on how decentralisation influences retention of rural health workers may benefit from the insight of health workers and people living in urban communities to identify and better understand pull and stay factors. Future studies on the relationship between decentralisation and retention of rural health workers should also include the perspectives of politicians and stakeholders beyond PHC, whose perspectives were not included in this analysis. Nonetheless, we believe that the findings discussed in this paper are valid, given that we triangulated our findings by conducting interviews and group discussions with different categories of stakeholders in different states, local governments, and rural communities.

## Conclusion

Because of the complex social, professional, and economic factors that influence the retention of health workers in rural areas, each setting requires a well-tailored and selected package of interventions from among those recommended by the WHO (4, 55). However, in countries where health system governance is weak, retention is as much a health-care human resources challenge as it is a health system governance issue. For example, our findings suggest that to improve retention of health workers in rural Nigeria, it may be necessary to ensure regular payment of salary, unify the salary scale of health workers across levels of care, and clearly define which tier of government takes primary responsibility for the salary of PHC workers. In addition, strengthening the engagement of rural communities in PHC can lead to increased retention of PHC workers in rural Nigeria, even in the presence of challenging working conditions. Therefore, interventions to improve retention of rural health workers in countries where health system governance is weak should take into account and seek to improve the weaknesses in health system governance, using both top-down and bottom-up strategies to improve the remuneration and support for health workers in rural communities.
