# Corrigendum

**DOI:** 10.3402/gha.v8.27839

**Published:** 2015-03-20

Regarding the paper titled: ‘How decentralisation influences the retention of primary health care workers in rural Nigeria’ by *Seye Abimbola, Titilope Olanipekun, Uchenna Igbokwe, Joel Negin, Stephen Jan, Alexandra Martiniuk, Nnenna Ihebuzor, Muyi Aina*

Published in *Global Health Action* (section “Original Articles”) 03 March 2015. Citation: Glob Health Action 2015, **8**: 26616 - http://dx.doi.org/10.3402/gha.v8.26616

In the Methods section, under the third sub-heading (Study participants), the third sentence is incorrect. It should read: In addition, we conducted IDIs with community members, and PHC managers working at local, state, and federal tiers of government (9 with community leaders, 8 with health committee members, and 15 with PHC managers).

This sentence currently reads:

In addition, we conducted IDIs with PHC workers, community members, and PHC managers working at local, state, and federal tiers of government (9 with community leaders, 8 with health committee members, and 15 with PHC managers).

The section should read:

## Study participants

The study participants were purposively selected to ensure that participants have the potential to provide rich, relevant, and diverse information on the research question. In each of six states, we conducted three FGDs with groups of PHC workers and three FGDs with groups of community members. In addition, we conducted IDIs with community members, and PHC managers working at local, state, and federal tiers of government (9 with community leaders, 8 with health committee members, and 15 with PHC managers). Each FGD involved 8–10 participants and lasted approximately 90 min, and each interview lasted approximately 60 min. We included as study participants all formal and full-time PHC workers involved in direct health-care provision such as nurses, midwives, community health workers, counsellors, and environmental health, laboratory and pharmacy personnel. Support staff such as cleaners and security guards were excluded, as were potential participants who could not communicate in any Nigerian language or who declined to sign consent forms. Given limited resources and time for this study, we also excluded all potential participants who were less than 18 years old because of logistic and ethical concerns associated with obtaining consent from and interviewing minors or involving them in group discussions.

